# Effectiveness of Telemonitoring for Respiratory and Systemic Symptoms of Asthma and COPD: A Narrative Review

**DOI:** 10.3390/life11111215

**Published:** 2021-11-10

**Authors:** Esther Metting, Lizayra Dassen, Jiska Aardoom, Anke Versluis, Niels Chavannes

**Affiliations:** 1 Data Science Center in Health, University of Groningen, University Medical Center Groningen, 9713 BZ Groningen, The Netherlands; 2 Department of Operations, Faculty of Economics and Business, University of Groningen, 9724 AV Groningen, The Netherlands; lizayrad@gmail.com; 3 National eHealth Living Lab, Medical Center, Leiden University, 2233 ZD Leiden, The Netherlands; j.j.aardoom@lumc.nl (J.A.); a.versluis@lumc.nl (A.V.); n.h.chavannes@lumc.nl (N.C.); 4 Public Health and Primary Care, Medical Center, Leiden University, 2233 ZD Leiden, The Netherlands

**Keywords:** telemonitoring, telehealth, telemedicine, asthma, COPD, respiratory symptoms, monitoring, eHealth, disease management

## Abstract

Asthma and chronic obstructive pulmonary diseases (COPD) are highly prevalent chronic lung diseases that require ongoing self-management, which itself is often suboptimal. Therefore, telemonitoring has been used to help patients measure their symptoms, share data with healthcare providers and receive education and feedback to improve disease management. In this study, we conducted a narrative review of recent evidence on the effectiveness of telemonitoring for asthma and COPD in adults. Of the thirteen identified studies, eleven focused on COPD and two focused on asthma. All studies were reviewed, and effects were compared between intervention and care as usual groups. Of the study interventions, seven showed a positive outcome on at least one outcome measure, and six had no significant results on any of the outcome measures. All of the interventions with a positive outcome included an educational component, while only one of the six interventions without positive outcomes included an educational component. We conclude that telemonitoring interventions for asthma and COPD seem more effective if they included an educational component regarding different aspects of self-management.

## 1. Introduction

Asthma and chronic obstructive pulmonary disease (COPD) are prevalent chronic pulmonary diseases requiring ongoing self-management. According to the World Health Organization, approximately 339 million people worldwide have asthma [1], and over 65 million suffer from moderate-to-severe COPD, making it the third leading cause of death worldwide [2,3]. Asthma typically starts early in life and is related to an allergy, whereas COPD is typically caused by air pollutants such as cigarette smoke or biomass fuel. Both diseases present with variable complaints and require treatment to reduce symptoms and prevent exacerbations (i.e., worsening symptoms and lung function).

Assessments by healthcare providers typically only offer a relatively static status of a patient at a given point in time and may not reflect their full range of symptoms and fluctuations. For example, in patients with asthma, it is not uncommon to have a normal lung function and no symptoms during the assessment, while being symptomatic at home [4]. Furthermore, patients may fail to recognize early signs of an exacerbation, leading to delays in consultation, diagnosis and treatment [5,6]. The early detection and intervention of an exacerbation can reduce recovery times and the need for hospitalization, while also improving quality of life (QoL) [6,7,8]. Frequent evaluations of symptoms and clinical parameters also facilitate personalized care, helping to enhance diagnostic accuracy, improve disease management and prevent exacerbations. However, healthcare providers already have a high workload [9], and increasing the number of clinical visits and assessments is undesirable.

Technological advancements have produced convenient and affordable tools for monitoring symptoms, including Bluetooth^®^ blood pressure devices, oximeters and mini spirometers. In addition, patients are increasingly able to access the internet, and healthcare providers and organizations are increasingly able to exchange medical data safely within specific digital environments. These developments have led to innovative possibilities for diagnosing, monitoring and treating patients with asthma or COPD. An example of this is telemonitoring. It allows patients to monitor their symptoms and physical parameters at home, share the data with healthcare providers and receive tailored treatment strategies based on that information. In this way, technology can support healthcare providers to deliver personalized disease management and more frequent symptom monitoring without the need for clinical visits or physical on-site assessments [10,11].

Telemonitoring can empower patients to become more actively involved in managing their asthma or COPD [12,13]. Numerous studies have shown that self-management is difficult and often poor in these groups, with an estimated 22–78% of patients having poor adherence to medical therapies [14]. Furthermore, incorrect inhaler technique is common [15], and 30–50% of symptomatic patients continue to smoke despite moderate-to-severe COPD [16]. Education can improve self-management skills and enhance disease control [17,18]. Thus, telemonitoring enables patients to be actively involved in their disease management and provides time-efficient education and feedback.

International asthma and COPD guidelines, such as the Global Initiative for *Asthma* (GINA) and the Global Initiative for Chronic Obstructive Lung Disease (GOLD) [19,20], acknowledge the potential of telemonitoring in disease management. Telemonitoring may offer benefits to disease status [21], health-related QoL (HR-QoL) [21,22], exacerbations [22], hospital admissions [22], exercise capacity [21,23] and healthcare utilization (including emergency room visits) [24]. To date, the considerable heterogeneity in the research methodology, monitoring devices, outcome variables and patient populations in studies of telemonitoring make it difficult to draw firm conclusions regarding its effectiveness [10,25,26] and feasibility [27] for these diseases. Implementing telemonitoring in healthcare can also be complicated by organizational limitations, technical matters and resistance from potential users [28]. Acceptance by stakeholders, integration in electronic health records and cost-effectiveness in comparison to current treatment are key to successful implementation. Many promising eHealth technologies have failed to realize their potential to improve outcomes due to resistance from healthcare providers or patients [28,29].

Telemonitoring has become more accessible for a large group of patients because the proportion of citizens with Internet access rises rapidly, and the elderly are increasingly in possession of smartphones. Moreover, more people have become digitally skilled [30]. Devices that measure vital signs, such as Bluetooth blood pressure devices, are readily available and can be linked with smartphone applications. An increasing number of healthcare organizations use Electronic Patient Records (EPR) that have the possibility of integrating with telemonitoring devices and applications such as Google or Apple Health. Moreover, an increasing number of healthcare insurance companies are starting to find ways to reimburse eHealth and telemonitoring. These advancements make the implementation of telemonitoring in daily clinical practice, nowadays, feasible for most healthcare organizations.

We aimed to conduct a narrative review of recent evidence comparing the effectiveness of care as usual with telemonitoring for symptoms (respiratory and systemic) of both asthma and COPD. The disease-related outcomes of interest are exacerbations, hospitalizations, HR-QoL and limitations in daily life. Information regarding implementation and feasibility is also assessed.

## 2. Materials and Methods

### 2.1. Eligibility Criteria

Only papers published in English in the past 5 years (i.e., 2016–2021), including adult patients with asthma or COPD were eligible for inclusion. Papers with multiple patient groups were included if the results for asthma and COPD were presented separately. The search revealed many papers that used telemonitoring for patients with different chronic diseases in the same paper, such as chronic heart failure (CHF), diabetes or COPD. Papers were only included if patients did not suffer from multiple chronic diseases (e.g., COPD or asthma with comorbid chronic heart failure, diabetes, etc.).

We included papers on telemonitoring interventions if they included active self-monitoring of respiratory symptoms and/or systemic symptoms (e.g., limitations in daily activities, lung function, cough, fatigue or weight), a comparison to care as usual (CAU) and reported outcome parameters according to GINA or GOLD guidelines. Only randomized controlled trials (RCTs) were included, but we did check the reference lists of overview articles (e.g., reviews and meta-analyses) to identify potentially eligible studies that had been missed in the initial search. All other research was excluded.

### 2.2. Literature Resources

The electronic databases of EMBASE, PubMed, MEDLINE and Web of Science were searched on the 17 August 2021. Endnote 20.0 was used to process the papers. To enhance comparability between studies and to help draw conclusions regarding the effectiveness of telemonitoring in the treatment of patients with asthma or COPD, we used the PICOS (Population, Intervention, Comparison, Outcome, Study design) framework to determine eligibility for inclusion. The search strategy for each database can be found in Appendix A. The chosen search terms were related to the study population (i.e., asthma or COPD), telemonitoring, and relevant outcome parameters.

### 2.3. Selection Procedure and Data Extraction

All papers were screened by one reviewer (EIM). All titles and abstracts were screened for eligibility before screening the full text of potentially eligible papers. Data extraction from the included studies was carried out by EIM, and the results were subsequently reviewed narratively.

## 3. Results

### 3.1. Selection of Papers

**Figure 1:**
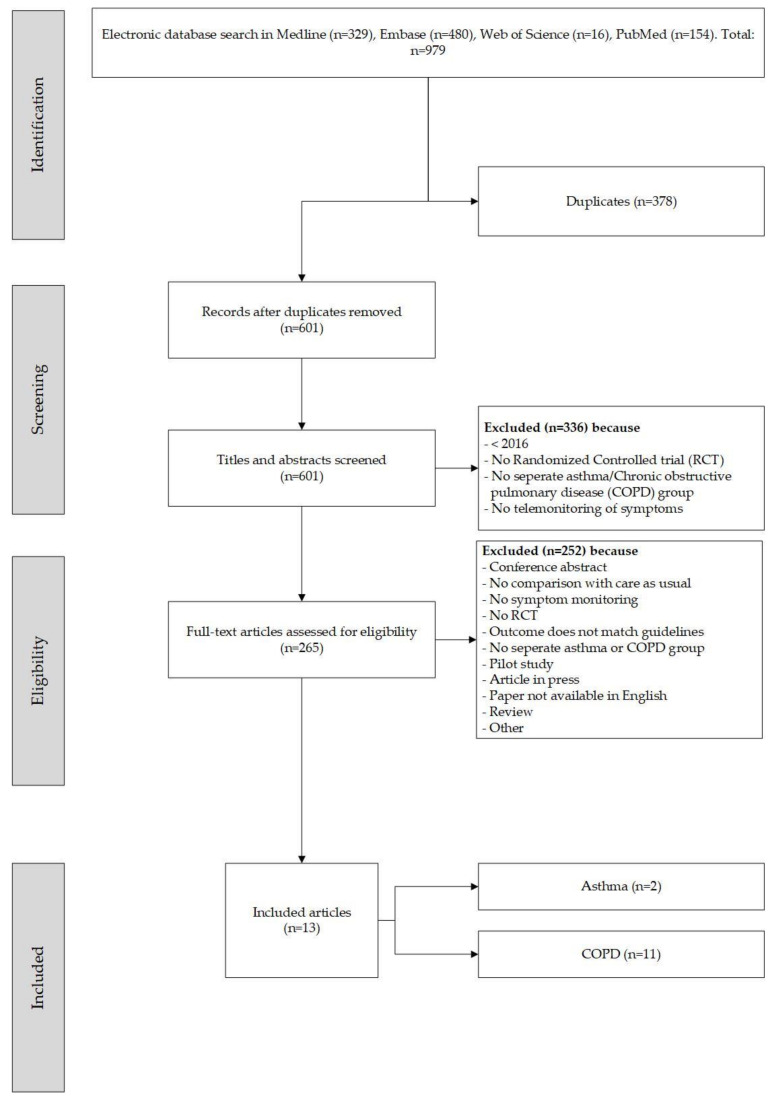
Flow diagram for literature identification and selection.

Figure 1 gives an overview of the literature search and selection procedure. The initial search identified 979 papers and, of these, 601 were screened based on their titles after removing duplicates. Next, the abstracts and/or full text of 265 potentially relevant papers were evaluated. In the end, 13 papers met the inclusion criteria and were included in the review.

### 3.2. Patient Characteristics

The characteristics of the included studies are presented in Table 1. Eleven studies included patients with COPD, and two papers included patients with asthma. The sample size across all the studies ranged from approximately 20 [31] to 250 [32] per study arm. Overall, five studies reported on the use of oxygen therapy (25% [33], 36% [34], 47% [35], 74% [36], 100% [37]). Baseline smoking status was reported in one of the asthma papers (13% current smokers) and in seven of the COPD papers (20–38% current smokers). Pack-years were reported in five COPD papers [31,34,36,38,39] and ranged from 40 to 56.

*Asthma studies:* the mean ages of the patients were 49 [40] and 50 years [41]; however, the age range in a study by Kim et al. [41] was broad (19–72 years). Approximately one-third [41] to half [40] of the study populations were male. The studies included patients with either moderate-to-severe [40] or fragile [41] asthma. In the former, this meant that patients were eligible if they had at least one asthma exacerbation in the past year that required an intensification of inhaled corticosteroid therapy. In the latter, no definition of fragile asthma was given. There was, thus, considerable variation in the baseline asthma severity. Although the mean Asthma Control Test (ACT) score was 22 (i.e., well controlled), it ranged from 7 to 25. Moreover, 43% of the patients received (increased) short-term systemic steroids to treat their exacerbation.

**Table 1:** Characteristics of the telemonitoring interventions.

	Platform for Patients	Monitoring Device(s)	Phone Monitoring	Structured Educational Component
ASTHMA STUDIES *				
Kim, M.-Y. et al. (2016).	Smartphone application with short message service (SMS) feedback	Smartphone application (snuCare), peak flow meter, symptom questionnaire in app	If the values require intervention	Automated personalized feedback and treatment support in the app based on an action plan
Nemanic, T. et al. (2019).	Web-based application or SMS	Peak expiratory flow device, online questionnaires	None	Patients were educated in guided self-management and on how to use an action plan
COPD STUDIES *				
Bernocchi, P. et al. (2018).	Phone calls by healthcare provider to collect information on disease status and symptoms	Pulse oximeter, portable electrocardiogram, pedometer	Weekly phone call to monitor disease status and symptoms	Project started with educational intervention
Ho, T.-W. et al. (2016).	Web-based electronic diary	Pulse oximeter, thermometer, BP meter. Electronic symptoms, vital signs and weight diary	If the values require intervention	Education after alert if the alert was considered innocent
Kessler, R. et al. (2018).	Telephone-based questionnaire and telephone/web platform	None	Weekly phone-based questionnaires to monitor symptoms	Self-management/coaching program “living well with COPD”
North, M. et al. (2020).	Online self-management app platform	MyCOPD app	Monthly phone calls to collect adverse events and CAT scores	Online self-management support app with “how to use the app” videos, and online education
Vasilopoulou, M. et al. (2017).	Tablet for exercises and secure web platform to collect data	Spirometry, heart rate meter, saturation 13 m, pedometer, tablet for questionnaires	Weekly phone call for dietary, psychological, and self-management advice	Training prior to the intervention on how to use devices
Ritchie, C. et al. (2016).	Interactive Discharge Assistant via phone calls	None	Phone calls with interactive voice response system to monitor symptoms and to provide customized patient education	Training prior to using devices and self-management intervention
Lilholt, P. H. et al. (2017).	Tablet that is connected to the devices	Telekit system with BP monitor, pulse oximeter, tablet, weight scale	None	None
Mínguez Clemente, P. et al. (2020).	Multiparametric recording unit that uploaded data to an online web platform	Pulse oximeter, portable electrocardiogram, BP gage, temperature and respiratory rate	If the values require intervention	None
Soriano, J. B. et al. (2018).	Electronic case report form	Pulse oximeter, BP gage, spirometer, respiratory rate and oxygen therapy compliance monitor		Training before the intervention on how to use devices
Stamenova, V. et al. (2020).	Cloud DX platform and connected Health Kit with tablet	Pulse wave wrist cuff monitor, oximeter, weighting scale, thermometer, tablet for questionnaires	Weekly feedback phone calls by the Respiratory Therapist	None
Walker, P. P. et al. (2018).	CHROMED (Clinical trials for Elderly Patients with Multiple Disease) monitoring platform	Within breath respiratory mechanical impedance	Monitoring of rescue medication, symptoms and QoL by phone	None

* Green: effective intervention, red: no effects.

*COPD studies:* the mean ages in these studies ranged from 63 [32] to 80 years [39]. The percentage of males was, on average, 42 [32] to 81% [33]. Ten studies included only patients with severe COPD, as defined by a previous exacerbation, hospitalization or poor lung function. In the remaining study [33], disease severity and previously experienced exacerbations were not inclusion criteria; however, 99% of the patients had ≥1 exacerbation in the past year. In addition, the average Assessment Test (CAT) score for the patients with COPD was indicative of marked symptoms. Furthermore, the forced expiratory volume (FEV_1_) ranged from 45 to 53%, indicating that the study [42] was comparable to the other COPD studies in terms of disease severity. Two of the eleven COPD studies included patients with comorbidities, more specifically congestive heart failure [32,35]. The data were presented separately for each patient group in both studies and could, therefore, be included in this study.

### 3.3. Intervention Characteristics

The included telemonitoring interventions were diverse in terms of the types of platforms used, monitoring procedures and devices and educational components (see Table 1). More specifically, in some interventions, the patients could share data with their healthcare providers on a website, and in others, this was via a tablet or smartphone. In this section, the characteristics of the interventions can be found.

*Asthma studies*: in one of the two asthma studies, the intervention was in an online environment. Both studies used provided further contact between the patients and the healthcare provider through an SMS (short message service) [40,41]. Within the asthma studies, the intervention times ranged from 8 weeks [41] to 12 months [39].

*COPD studies*: In three of the eleven studies, (automated) phone calls were used to monitor disease severity [32,33,43]. All the COPD studies compared telemonitoring with CAU. Information regarding the CAU procedure was hardly described in the papers of North et al. [44], Soriano et al. and Ho et al. [39]. In a study by Lilholt et al., the control patients were treated and monitored by their GP and received the telemonitoring intervention after the study period [45]. A study by Clemente et al. [34] was an exception, as they clearly described the frequency and goals of the home hospitalization and discharge procedure without telemonitoring. Both groups received health education and the patients’ informal carers were included in the procedure.

This was not the only study where control patients received education; Bernocchi et al. [46] described that patients receiving care as usual were provided with an educational session on healthy lifestyle and were invited to daily physical activity practices. In the COMET study, education was provided to control patients but this depended on the clinical center specific procedure [36]. Usual care in a study by Ritchie et al. [32] consisted of the provision of discharge instructions regarding lifestyle, follow up, monitoring and medication. Some patients received additional support from social work or home health services.

Stamenova et al. [43] and Vasilopoulou et al. [33] compared two intervention conditions with CAU. In the first study, the following two intervention groups monitored their symptoms and clinical parameters in a COPD clinic: (1) a self-management group that did not have their data actively monitored by the clinic, and (2) a remote monitoring group that had the clinical project specialist call patients every week for health evaluation and education [43]. Moreover, data monitoring was active in the remote monitoring group, with action taken when values exceeded predefined thresholds. In the second study, all the patients assigned to an intervention group attended a hospital outpatient rehabilitation program for 2 months. After that, one group received maintenance rehabilitation as an outpatient, and one group received maintenance telerehabilitation at home, both for twelve months. Throughout the 14-month study, a third group received CAU that consisted of optimal pharmacotherapy, oxygen therapy if needed, vaccinations, regular follow-up assessments by a pulmonologist and training to timely recognize exacerbations. This group did not participate in the initial two-month rehabilitation program [33].

### 3.4. Outcome Measurements and Effects of the Intervention

#### 3.4.1. Parameters Used

See Table 2 for an overview of the primary and secondary outcomes, comprising a wide variety of parameters.

#### 3.4.2. Effects of the Intervention

The wide variation of outcome parameters challenges the comparison of results across studies. Some studies evaluated only patient-related outcomes, and others also included process parameters such as feasibility or QALYs. Below, the reported effects of the studies are presented.

*Asthma studies.* Telemonitoring positively affected medication adherence in one of the asthma studies [41]. Adherence and quality of life improved after eight weeks in the patients who used the application to record their symptoms, and peak expiratory flow twice a day improved, whereas the control group did not improve on either of the parameters. There was no change in the lung function or exacerbation rates between the groups. The other asthma study [40], with a follow-up time of 12 months, showed a statistical improvement in asthma control in both groups, but this was not clinically relevant. A sub-analysis showed that the patients with two or more exacerbations in the past year showed an improvement in asthma control after 12 months, whereas the control group showed no improvement. There was no intervention effect on lung function in this study.

*COPD studies.* Among the papers evaluated, eight of the eleven COPD studies showed positive results for the intervention, and five showed no significant results [34,37,38,43,45]. In a study by Stamanova et al., the adherence rate of patients was high, despite the negative effects. In this study, all the groups, including the control group receiving care as usual, improved in self-efficacy and disease knowledge, despite the fact that no educational intervention was provided [43]. Liholt et al. [45] measured Health Related Quality of Life (HR-QoL) and found no difference between the control and intervention group, despite the large sample of 1225 patients. Patients in a study by Walker et al. [38] were highly compliant with the intervention, and a wide range of parameters was evaluated. However, no difference was found between the control and the intervention group in the parameters they planned to assess. Additional analysis showed that the average hospital duration stay in the intervention group was shorter than in the control group (control group: 4 days, intervention: 1 day, *p* = 0.045). In addition, the intervention group in this study was less likely to be re-hospitalized (incidence ratio 0.46, *p* = 0.002).

In contrast, patients in a study by Soriano et al. [37] showed no difference on any parameter compared to usual care. Despite this, the patients were very satisfied, all the patients would recommend the telemonitoring system to others. Furthermore, 93% of the physicians would use the system again when necessary. Similarly, another study [34] also showed no improvement in the primary and secondary outcomes, even though adherence to the study was very high (no dropouts) and the intervention patients were satisfied with the procedure. Although there was no clinical improvement, an important outcome of the study was that the number of healthcare staff visits could be reduced. This did not lead to a reduction in healthcare costs.

Telemonitoring positively influenced exercise tolerance in a study by Bernocchi et al. [35]. COPD patients received an educational intervention consisting of weekly phone calls to collect data and provide self-management advice. The patients self-monitored their vital symptoms, physical activity and used a diary. The intervention group improved more than the control group on the 6 min walk test, number of hospitalizations, time to event, quality of life, impairment/disability and dyspnea severity.

The my COPD app study led to improvements in inhaler technique, exacerbations, hospital readmissions and COPD health status [31]. In the app, patients filled in their symptoms, medication and the COPD Assessment Test questionnaire (CAT). The app provided an educational program based on the input. However, only 40% of the patients were actively using the app until the end of the study. No improvements were found in activation, anxiety, depression and dyspnea.

In a study by Kessler et al. [36], the intervention group improved more than the control group in unplanned acute ward visits and hospitalizations. The BODE (Body mass index, airflow obstruction, dyspnea and exercise) index and mortality rate were better in the intervention group. Interestingly, 26% of the patients quit smoking in the intervention group compared to 6% in the control group. The patients in the intervention group received a program that included self-management intervention, home monitoring and access to an eHealth telephone/web platform on which they received weekly health status information. This study showed no improvements in the 6 min walking test, exacerbations and depression/anxiety.

In the E-Coach study [32], patients started with the intervention when hospitalized and received support from a care transition nurse and an interactive voice response system. This approach led to fewer hospitalization days and improved community tenure in the intervention group but did not improve rehospitalization rates or death rates. According to the researchers, the reason for the improvement in community tenure is unclear and required future research.

A study by Ho et al. [39] showed that an intervention where patients monitored their vital and respiratory symptoms in combination with a diary led to an increased time to readmission for a COPD exacerbation and reduced ER visits. However, it did not improve the number of COPD readmissions and COPD exacerbations. Finally, Vasilopoulou et al. [33] compared three patients groups; the telemonitoring group and the home monitoring group (without eHealth) both improved in COPD exacerbations and hospitalizations compared to a control group without structural monitoring.

### 3.5. Integration of the Telemonitoring Programs in the Healthcare Organization

#### 3.5.1. Feasibility and Safety

*Asthma studies.* Kim et al. [41] concluded that their intervention was feasible, reporting that patients were generally satisfied, that “ease to use” received the highest score, and that 23% of the patients considered the application (somewhat) helpful [41]. Nemanic et al. [40] reported that 78% of patients had at least one subjective positive effect and that 80% would continue the intervention. Feasibility and safety were not measured directly, but 98% completed the 12-month study [40].

*COPD studies.* Feasibility and/or safety was assessed in four of the eleven studies. These indicated that telemonitoring interventions produced no major side effects [34,35,36] and were feasible [31,35,36]. In most, data were sent automatically to a database of the healthcare organization. Healthcare providers received an automated warning or red flag at defined thresholds or when data collection stopped [32,34,38,45,47]. In one of the studies, the patient also received a warning by email [43]. Several interventions granted patients access to a phone number that could be used for emergencies, coaching or advice [32,33,35,39,43]. Soriano et al. [37] made a distinction between technical and clinical alerts but did not describe the purpose of these alerts [37]. Ho et al. [39] reported that 57% of the 192 alerts from 40 patients required a phone consultation. The remaining alerts were considered minor, requiring only health education, advice, observation or reassurance [39]. Moderate to high satisfaction with the intervention group was reported in the only study to measure this metric [41]. Only one study described the level of integration in the healthcare system, reporting that it was not fully integrated with the medical health records system [32].

#### 3.5.2. Acceptability and Adherence

*Asthma studies.* In both asthma studies, patients were satisfied with the intervention, and compliance was high [40,41]. Appreciation for the intervention was highest when the intervention was easy to use or user-friendly [41]. Patients reported that the intervention had improved their symptoms and they would like to continue [40].

*COPD studies.* Only a few papers on COPD provided information about acceptability and adherence. Ritchie et al. [32] reported that one-third of their patients answered all seven automated phone surveys in the first week, 85% answered all the surveys, and the care transition nurses performed almost five calls per patient for so-called “red flags” during the intervention. All but one of the studies evaluating adherence have shown high usage rates, as 93% of the patients reportedly performed the prescribed exercises [35] and almost 94% of the patients were compliant with all the monitoring [33]. A study by Liholt et al. showed no result of the intervention and a high attrition rate (52% lost to follow-up) [45].

### 3.6. Education and Self-Management

All the interventions with at least one beneficial effect on one or more parameters included some form of patient education before and/or during the intervention, although the approach varied between the studies. The asthma studies provided guided self-management and instructions on how to use an asthma action plan [40], as well as feedback on self-management [41]. The COPD studies included training to use the study equipment [33,39], disease management advice and exercise instruction in weekly calls [35], education on warning signs and symptoms [32] and app-based education (e.g., inhaler technique videos) [31]. Among the ineffective interventions (only COPD), only one provided some form of education [37], with the remaining four requiring that patients simply monitor symptoms and/or vital signs. Finally, Kessler et al. showed that the beneficial effect of intervention was only present in patients who attended at least 25% of the planned coaching sessions [36]. A complete overview of the studies is presented in Appendix B.

## 4. Discussion

### 4.1. Main Results

This narrative review summarized the findings of thirteen RCTs of telemonitoring interventions for asthma (n = 2) and COPD (n = 11). Eight showed clinical improvements, mainly regarding (time to) exacerbations, hospitalizations or death, and three of these demonstrated symptom improvement. Approximately one-third of the studies also evaluated safety and feasibility, and these all showed that the interventions were feasible and free of adverse events. When the monitored symptoms exceeded a certain threshold, healthcare providers in all the studies received automated warnings, and if needed, patients were called for further intervention. Despite the strict inclusion criteria, there was still large variation in the number of patients, the interventions, the follow-up times and the outcome measurements among the studies. However, the main difference between effective and ineffective interventions seemed to be the inclusion of some form of patient education in all the effective interventions compared to one-fifth of the ineffective interventions. Whether the improvements were caused by the educational intervention alone or the combination of telemonitoring and education (and possibly other factors) remains to be elucidated

### 4.2. Comparison with Current Literature

Recent systematic reviews and meta-analyses on telemonitoring in asthma or COPD have shown that negative effects on clinical outcomes are rare [48], consistent with our finding of either positive outcomes or similar effects in comparison to usual care. Furthermore, feasibility and safety were also assessed in some of the included studies, revealing no adverse effects. Studies have also shown that telemonitoring can be feasible and acceptable for older people with COPD. Thus, we conclude that telemonitoring seems to be a safe and promising approach to support disease management in patients with asthma and COPD.

#### 4.2.1. Telemonitoring and Patient Education

Most telemonitoring interventions with at least one positive outcome had integrated an interactive educational component. Hong and Lee [22] previously found a similar effect in a meta-analysis of telemonitoring for patients with COPD. Active patient involvement through education or skills delivery to support coping with the disease seems to improve the outcomes. A reason for this mediating effect might be that telemonitoring is dependent on behavioral change in the patient and healthcare provider. It is important for patients to follow the monitoring instruction and for healthcare providers to use the results of the monitoring in their management, and using telehealth to deliver education can empower patients by giving them greater insight and the tools to manage their disease [10]. Bonnevie et al. [21] showed that interventions with automated feedback, representing a form of patient education, improved long-term adherence to home-based exercise therapy. Enhanced self-management can improve physical activity, avoidance and medication adherence. This could explain the greater effectiveness of telemonitoring programs with an educational component.

#### 4.2.2. Accetability, Feasibility and Adherence

The effectiveness of telemonitoring applications on disease outcomes was evaluated in the studies evaluated in this review, with positive effects only found in some. It may be that there is no direct link between the telemonitoring intervention and disease outcomes. For example, if the application is not used (correctly) by the patient or if the healthcare provider is not using the collected data, health status will not be affected by telemonitoring alone. Instead, behavioral and implementation factors likely moderate the effectiveness of any intervention, which makes it remarkable that these are rarely measured in telemonitoring effectiveness studies. Some papers only described the feasibility of the intervention or the satisfaction with the program, failing to mention the behavioral and implementation factors that will also affect the results of telemonitoring. If the patient does not use an intervention, or if it is not correctly implemented in the healthcare process, it cannot be effective. To improve telemonitoring adherence and implementation for asthma or COPD management, greater attention should be given to patient behavior and user-friendliness. Furthermore, it remains unclear if and how healthcare providers used the telemonitoring results in clinical decisions, and indeed if patients’ self-management improved due to symptom monitoring. These uncertainties limit our ability to pinpoint which moderating or mediating factors led to the observed clinical effects in the included studies.

Interventions with similar clinical effects to CAU may still be relevant if they improve other parameters, such as indirect costs, e.g., workload, work satisfaction or time and travel burdens. Michael Porter proposed the concept of value-based healthcare [49] to support decision making in healthcare by weighing the following three integrated concepts: patients value, health outcomes and costs. This suggests that implementing a telemonitoring innovation can be of value if health outcomes remain stable and patient satisfaction and/or costs improve. Unfortunately, the included studies merely focused on clinical outcomes, which may have led to the unnecessary rejection of interventions that improve value-based healthcare. There is an urgent need for studies that assess all the concepts related to healthcare improvement, not merely clinical effects, for telemonitoring interventions.

### 4.3. Strengths and Limitations

The COVID-19 pandemic stressed the importance of studies about telemonitoring [50]. Due to the high infection rates of COVID-19, chronic and vulnerable patients were not able to attend regular clinical assessments, and spirometry was not taken during the outbreak of the virus. By applying telemonitoring, patients can receive care at home without risk of becoming infected. Papers such as this one can support telemonitoring developers and healthcare organizations to tailor interventions toward the requirements of patients. The timing of this publication is, therefore, one of the strengths of this study. Another strength of this study is that we only included recent RCTs. This ensures stronger and more topical evidence regarding the effectiveness of telemonitoring than might be obtained from older data or that obtained from observational and cohort studies. We also took into account factors related to acceptability, adherence, feasibility and safety, which are key aspects of practical use and future implementation in healthcare organizations. Moreover, we only included interventions where the patient was actively involved in the monitoring process. This strengthens the insights on issues patients or users face when using telemonitoring and offers a broader and more complete analysis of the effectiveness of telemonitoring.

Aside from strengths, the review had some important limitations. First, the strict inclusion criteria meant that only a limited number of publications could be included. Secondly, there was wide variation in terms of study design, participant characteristics and outcome variables. This made the results difficult to compare across the studies and may have reduced the reliability of our conclusions, limiting the generalization of results. Finally, the initial screening and review of the papers was conducted by one author, which may have introduced bias.

### 4.4. Future Research

Most included studies were performed for COPD, with a limited number of studies focusing on asthma. This finding is remarkable given the prevalence of asthma and its significant adverse impact on HR-QoL. Thus, more studies of the impact of telemonitoring on asthma are urgently needed. Future research should also analyze the roles of telemonitoring for groups with different severities of asthma or COPD to explore if this is a determinant of the effectiveness and feasibility of telemonitoring. Primary care offers an ideal setting for such research.

Information regarding how exactly telemonitoring was implemented in healthcare organizations was hardly mentioned in the studies. For instance, it was unclear whether healthcare professionals could see patient’s data in their EPR, if and how the alerts were used and if a healthcare insurance company covered the intervention costs. Implementation factors are important because they provide information to readers regarding the feasibility of applying the approach in their own medical practice. It is important to obtain more insight into how telemonitoring can be embedded in healthcare systems, e.g., how costs can be reimbursed, how data can be integrated in the EPRs and which devices are suited.

It is also important to gain better insight into the behavioral and implementation factors that mediate or moderate the effectiveness of telemonitoring on clinical outcomes. Poor knowledge of these concepts might be responsible for the inconclusive outcomes of many studies to date; researchers may need to cast a wider net, beyond traditional intervention and clinical effects alone, ensuring that they also include behavioral and implementation concepts.

This review showed that patient education positively affected the clinical outcomes of telemonitoring interventions, but it did not find an explanation as to why this happens. Future studies ought to look at the interaction between telemonitoring and education to better understand the working mechanisms. It is equally important to determine if positive effects are caused by education alone, or by combining telemonitoring with education.

## 5. Conclusions

Telemonitoring is effective, feasible and safe compared to care as usual for patients with COPD. There was an insufficient number of studies to draw conclusions regarding asthma telemonitoring. Telemonitoring can improve several clinical outcomes in COPD patients, including the need for hospitalization, length of hospitalization, number of clinical visits, QoL and number of exacerbations. Adding an educational element to a telemonitoring intervention seems to increase the prospect of a positive effect. However, there is a lack of research on the behavioral and process factors related to telemonitoring. Future research should focus on the effects of telemonitoring in patients with asthma, the full telemonitoring process for the patient and the healthcare provider and its implementation in the healthcare organization, as well as the impact of patient and healthcare provider characteristics.

## Figures and Tables

**Table 2:** Primary and secondary outcomes of telemonitoring interventions for patients with asthma or COPD.

Asthma				COPD			
Primary	Reference	Secondary	Reference	Primary	Reference	Secondary	Reference
Feasibility	[40,41]	Medication adherence	[41]	Exercise tolerance	[35]	Physical activity	[33]
Asthma Control	[40]	Asthma health status	[40]	Time to hospitalization or Hospitalization duration	[32,36,38,39]	HR-QoL	[33,38]
		Events *	[40]	HR-QoL	[45]	Healthcare usage	[43]
				Exacerbation frequency	[33]	QALYs	[38]
				COPD health status	[31]	BODE index	[36]
				COPD health status	[31]	Smoking cessation	[43]
						Events *	[36]
						Dyspnea	[35]
						Slower lung function decline	[33]
						Time to event	[32,34,36,38,39,43]
						COPD Health status	[43]

Abbreviations: COPD, chronic obstructive pulmonary disease; HR-QoL, health-related quality of life; QALYs, quality adjusted life-years. * Events included exacerbations, mortality and hospitalizations.

## Abbreviations

ACT, Asthma Control Test; CAT, COPD Assessment Test; CAU, Care as usual; CHF, chronic heart failure; COPD, chronic obstructive pulmonary disease; ECG, electrocardiogram; eNO, exhaled Nitric Oxide; FEV1, Forced Expiratory Volume; FVC, Forced Vital Capacity; HR-QoL, Health-related Quality of Life; MLHFQ, Minnesota Living with Heart Failure Questionnaire; MRC, Medical Research Council scale; PASE, Physical Activity Scale for the Elderly; QoL, Quality of Life.

## Appendix A. Search Terms

PubMed, 17 August 2021, 154 results, 17 August 2021 (“2016/01/01”[Date–Publication]: “3000” [Date–Publication]) AND (“asthma chronic obstructive pulmonary disease overlap syndrome”[MeSH Terms]) OR (copd[Title/Abstract]) OR (asthma[Title/Abstract]) AND (telemedicine[MeSH Terms]) AND (disease exacerbation[MeSH Terms]) OR (administration, hospital[MeSH Terms]) OR (activities of daily living[MeSH Terms]) OR (quality of life[MeSH Terms]).

Web of Science, 17 August 2021, 248 results, 17 August 2021. AB = (asthma OR COPD OR “Chronic Obstructive Pulmonary disease” OR ACO OR “asthma chronic obstructive pulmonary disease overlap syndrome” OR “Overlap syndrome”) AND AB = (telemonitoring OR telemedicine OR telehealth OR telerehabilitation) AND AB = (exacerbation OR “Quality of life” OR “health related quality of life” OR hospitali *ation * OR limitation OR activities).

EMBASE (804 results) and Medline (582 results), 17 August 2021. Now without comparison with care as usual: (‘asthma’/exp OR ‘asthma’ OR ‘asthma bronchiale’ OR ‘asthma pulmonale’ OR ‘asthma, bronchial’ OR ‘asthmatic’ OR ‘asthmatic subject’ OR ‘bronchial asthma’ OR ‘bronchus asthma’ OR ‘childhood asthma’ OR ‘chronic asthma’ OR ‘lung allergy’ OR ‘chronic obstructive lung disease’/exp OR ‘chronic airflow obstruction’ OR ‘chronic airway obstruction’ OR ‘chronic obstructive bronchopulmonary disease’ OR ‘chronic obstructive lung disease’ OR ‘chronic obstructive lung disorder’ OR ‘chronic obstructive pulmonary disease’ OR ‘chronic obstructive pulmonary disorder’ OR ‘chronic obstructive respiratory disease’ OR ‘chronic pulmonary obstructive disease’ OR ‘chronic pulmonary obstructive disorder’ OR ‘copd’ OR ‘lung chronic obstructive disease’ OR ‘lung disease, chronic obstructive’ OR ‘obstructive chronic lung disease’ OR ‘obstructive chronic pulmonary disease’ OR ‘obstructive lung disease, chronic’ OR ‘pulmonary disease, chronic obstructive’ OR ‘pulmonary disorder, chronic obstructive’ OR ‘asthma-chronic obstructive pulmonary disease overlap syndrome’/exp OR ‘asthma-chronic obstructive pulmonary disease overlap syndrome’ OR ‘asthma-copd overlap syndrome’ OR ‘asthma chronic obstructive lung disease overlap syndrome’/exp OR ‘asthma chronic obstructive lung disease overlap’/exp) AND (‘telemonitoring’/exp OR ‘distant monitoring (patient)’ OR ‘distant patient monitoring’ OR ‘remote monitoring (patient)’ OR ‘remote patient monitoring’ OR ‘tele monitoring’ OR ‘telemonitoring’ OR ‘telemedicine’/exp OR ‘tele medicine’ OR ‘telemedicine’ OR ‘telerehabilitation’/exp OR ‘e-rehabilitation’ OR ‘remote rehabilitation’ OR ‘tele-rehabilitation’ OR ‘telerehabilitation’ OR ‘virtual rehabilitation’ OR ‘telehealth’/exp) AND (‘disease exacerbation’/exp OR ‘aggravation, disease’ OR ‘disease aggravation’ OR ‘disease exacerbation’ OR ‘disease flare’ OR ‘disease progression’ OR ‘exacerbation, disease’ OR ‘exacerbation’/exp OR ‘exacerbations of chronic pulmonary disease tool’/exp OR hospitalization OR ‘hospitalization’/exp OR ‘hospital stay’ OR ‘hospitalization’ OR ‘short stay hospitalization’ OR ‘quality of life’/exp OR ‘hrql’ OR ‘health related quality of life’ OR ‘life quality’ OR ‘quality of life’ OR ‘daily life activity’/exp OR ‘adl (activities of daily living)’ OR ‘activities of daily living’ OR ‘activity, daily living’ OR ‘daily life activity’ OR ‘daily living activity’ OR ‘activity of daily living assessment’/exp OR ‘activity of daily living assessment’ OR ‘daily life activity assessment’).

## Appendix B. Study Characteristics and Intervention Effects

**Table:**

Authors	Population	Selection	Aim	Telemonitoring	Comparison	Effect	Study Design	Follow-Up	Additional Support	Acceptability and Feasibility
ASTHMA STUDIES										
Kim, M.-Y. et al. (2016).	I (n = 22), 18% male, mean age 49 (19–72), C (n = 22), 36% male, mean age 51 (34–67) years.	Asthma patients >19 years	Explore feasibility and effectiveness of the snuCare in adult patients with asthma.	snuCare app with peak flow meter, daily symptom scores. App provided feedback based on action plan.	1:1 CAU.	Enhanced medication adherence Intervention group: *p* = 0.017, control group *p* = 0.571. FEV_1_, asthma control and QoL did not improve.	Random controlled trial.	8 weeks	3 visits. Researchers received automatic warnings through the system if measurements were worrisome.	Application was deemed feasible to use and was effective.
Nemanic, T. et al. (2019).	I (N = 51), 47% male, mean age 45 (39–61) years. C (n = 49), 49% male, mean age 53 (40–60) years.	Confirmed asthma diagnosis, ≥1 exacerbation last year or symptoms more than twice a week and activity limitation	To test the influence of telemonitoring on asthma control, exacerbation rate and severity.	Via SMS or webportal: ACT, pulmonary function tests, eNO, questionnaire data on knowledge, compliance, symptoms, exacerbations.	1:1 CAU.	No difference between control and intervention group in asthma control change for patients with moderate asthma symptoms. For severe patients with asthma (i.e., patients with two or more exacerbations prior to study inclusion), a significant increase in ACT was detected.	Single center prospective randomized controlled trial.	12 months	Study nurse received automatic warning if values are worrisome.	Home monitoring in asthma is feasible and effective for patients with more severe symptoms.
COPD STUDIES										
Bernocchi, P. et al. (2018).	I: 88% male, 71 ± 9 years, C: 75% male, 70 ± 10 years.	Patients with COPD (GOLD B, C, D) and CHF undergoing hospital rehabilitation with ≥6 months life expectancy	Feasibility and effectiveness of intervention on exercise tolerance and time to event hospitalization, death, dyspnea, physical activity, disability and QoL.	Weekly phone call to assess symptoms and disease status. Pulse oximeter, portable ECG, mini ergo meter, pedometer, diary.	1:1 CAU.	After 4 months change in Δ6MWT: I 60, C −15 (*p* = 0.004) in favor of I. Time to hospitalization/death: I 113 days, C 105 days (*p* = 0.0484). I improved more than C in disease status: ΔMRC, ΔPASE, ΔBarthel, ΔMLHFQ and ΔCAT.	Randomized controlled trial.	4 months	Intervention group received weekly monitoring calls from nurse tutor within addition educational and motivational input.	Intervention is feasible and safe. No major side effects observed.
Ho, T.-W. et al. (2016).	I (n = 53), C (n = 53). 76% male, mean age 80 ± 9 years.	COPD (FEV_1_/FVC < 70%) patients discharged after exacerbation, current or former smokers	Reduce the frequency of readmission.	Pulse oximeter, thermometer, blood pressure meter, online symptom diary.	1:1 CAU.	Number of all cause exacerbation episodes/patient improved. Hospital admissions I: 0.23, C: 0.68 (*p* = 0.002). Emergency room visits I: 0.36, C: 1.29 (*p* = 0.006).	Randomized controlled trial.	6 months	Healthcare providers receive notification if values are concerning according to algorithm. Patients in both groups had access to medical counseling via phoneline.	
Kessler, R. et al. (2018).	I (n = 172), 69% male, mean age 67 ± 9 years. C (n = 173), 69% male, mean age 67 ± 9 years.	Patients with COPD (post bronchodilator FEV_1_/FVC < 70% and FEV_1_ < 50%), ≥ 10 pack-years, ≥ 1 severe exacerbation in the past year, ≥ 6 months life expectancy	Reduce hospitalization, reduce length of stay and improve patients coping behaviors.	Weekly/daily symptom monitoring, spirometry, pulse oximeter, heart rate. Patients on long-term oxygen therapy were monitored with NOWOX.	1:1 CAU.	Intervention: 23% fewer all cause hospitalization days (*p* = 0.047), BODE index (Body mass index, Obstruction, Dyspnea, Exercise capacity). After 12 months 0.8 points lower in intervention group (*p* = 0.010), less mortality in Intervention group (1.9% vs. 14.2%, *p* < 0.001).	International Randomized controlled clinical trial.	12 months	Study performed in France, Germany, Italy and Spain; 3 monthly hospital visits and regular phone call by hospital staff.	Safety was assessed and intervention was safe.
Lilholt, P. H. et al. (2017).	I (n = 578), 48% male, mean age 70 ± 9 years. C (n = 647), 44% male, mean age 70 ± 10 years.	Patients with COPD with CAT ≥ 10 or MRC ≥3 or mMRC ≥ 2 or ≥ 2 exacerbations in the past 12 months	To assess the effect of telehealthcare on HR-QoL.	Telekit system with tablet, blood pressure monitor, pulse oximeter, health precision scale.	1:1 CAU.	No HR-QoL improvement in either intervention or control group.	Pragmatic cluster randomized trial.	12 months	High attrition rate (52% lost to follow-up). Patients were contacted if measurements were not performed or is values were worrisome.	Telehealthcare was deemed feasible.
Mínguez Clemente, P. et al. (2020).	I (n = 49), 77% male, mean age 68 ± 8 years. C (n = 52), 60% male, mean age 70 ± 8 years.	patients with COPD admitted for exacerbation. Clinical stabilization in 4 days **	Effectiveness of telemonitoring on time until first exacerbation post discharge.	Temperature, Blood pressure, respiratory rate, Oxygen saturation, heart rate, ECG.	Traditional follow-up based on daily visits.	No HR-QoL improvement in either intervention or control group. However, intervention group had the same results with less home visits compared to control group.	Randomized controlled trial.	6 months	Patients received telephone calls from the physician to evaluate clinical situation and actions. If values were worrisome, the physician received an SMS warning.	
North, M. et al. (2020).	I (n = 20), 65% male, mean age 65 ± 6 years. C (n = 21), 52% male, mean age 68 ± 7 years.	patients with COPD using an inhaler, current or ex-smoker	Evaluation of safety and effectiveness of myCOPD.	CAT evaluation every 4 weeks.	1:1 CAU.	The treatment effect on the CAT score was 4.49 (95% CI: −8.41, −0.58) points lower in the myCOPD arm. Patients’ inhaler technique improved in the digital intervention arm (101 improving to 20 critical errors) compared to usual care (100 to 72 critical errors). Exacerbations tended to be less frequent in the digital arm compared to usual care; 18 vs. 34 events. Hospital readmissions risk was numerically lower in the digital intervention arm: OR for readmission 0.383 (95% CI: 0.074, 1.987; n = 35).	Parallel arm feasibility randomized controlled trial with blinded outcome assessment.	90 days	All patients were contacted by phone at 30, 60 and 90 days to record symptom data. About 50% of the eligible patients declined to participate. Authors did not report *p*-values.	The use of digital platforms in patients with COPD is feasible.
Ritchie, C. et al. (2016).	patients with COPD: I (n = 65), 42% male, mean age 64 ± 11 years. C (n = 67), 69% male, mean age 63 ± 11 years.	Hospitalized COPD/CHF patients who are expected to be discharged, ≥ 6 months life expectancy. Patients with impaired cognition could participate of a caregiver could serve as proxy	Evaluation of the eCoach effectiveness on rehospitalization, mortality and measure of community tenure.	eCoach: automated calls from the interactive discharge assistant for risk assessment.	1:1 CAU.	In the COPD subgroup the intervention was related to fewer hospital days after 30 days compared to control (0.5 vs. 1.6, *p* = 0.03).	Pragmatic randomized trial.	90 days	Healthcare providers had access to dashboard to review the data.	
Soriano, J. B. et al. (2018).	I (n = 115), C (n = 114). Total: 80% male, mean age 71 ± 8 years.	patients with COPD 50–90 years, with FEV1 <50%, treated with chronic home oxygen therapy, ≥2 exacerbations past year, currently clinically stable	Estimate effectiveness of intervention on exacerbations leading to emergency department visits/hospital admissions with telehealth.	Modem, pulse oximeter, blood pressure gage, spirometer, respiratory rate and oxygen therapy compliance monitor.	1:1 CAU.	No effect on exacerbations or hospitalizations.	Multicenter, nonblind, randomized controlled trial.	12 months	No additional support. Monitoring Center for healthcare provider with traffic light indicator.	Patients and doctors were satisfied with the program.
Stamenova, V. et al. (2020).	Self-monitoring (n = 41), 56% male, mean age 72 ± 7 years. Remote monitoring (n = 41), 56% male, mean age 72 ± 10 years. CAU (n = 40), 52% male, 73 ± 9 years.	COPD diagnosis	Evaluate effectiveness of technology enables self-management program with remote monitoring and CAU.	Bluetooth enabled device kit for oxygen saturation, blood pressure, temperature, weight, symptoms.	2 intervention groups, 1 CAU group.	No difference in self-efficacy, disease knowledge or disease severity between the groups. No changes compared to baseline in symptoms or activity scores in any of the groups. No differences in healthcare utilization, emergency room visits, hospital admissions or healthcare visits.	3 arm randomized controlled trial.	6 months	Weekly evaluation and education phone calls. All patients could email or call the clinic with non-urgent questions. Clinical project specialist received automatic warning if values are worrisome.	
Vasilopoulou, M. et al. (2017).	C (n = 50), 74%, mean age 64 ± 8 years. Tele-rehabilitation (n = 50), 88% male, mean age 67 ± 10 years. Hospital-based rehabilitation (n = 50), 76% male, 67 ± 7 years.	patients with COPD, with FEV1 <80%, optimal medical treatment without regular use of OCS, ≥1 exacerbation past year	Evaluate the effectiveness of regular home monitoring of vital signs combined with teleconsultation sessions on acute exacerbations, hospitalizations and emergency department (ED) visits.	Tablet and heart rate monitor, pulse oximeter, symptoms, pedometer, spirometry, oximetry, HR-QoL, CAT, Hospital Anxiety and Depression Scale (HADS), mMRC.	3 groups: ^(1)^ CAU, ^(2)^ 2-month outpatient rehabilitation and home maintenance tele-rehabilitation and ^(3)^ 2-month outpatient rehabilitation and hospital maintenance rehabilitation.	Home-based tele-rehabilitation and hospital-based pulmonary rehabilitation remained independent predictors of a lower risk for ^(1)^ acute exacerbation: IRR 0.52, 95% CI 0.39–0.69, and IRR 0.64, 95% CI 0.47–0.85), and ^(2)^ hospitalization: IRR 0.19, 95% CI 0.10–0.36, and IRR 0.38, 95% CI 0.21–0.68). Only home-based maintenance tele-rehabilitation was an independent predictor of reduced ED visits (IRR 0.12, 95% CI 0.07–0.19).	Prospective randomized controlled trial.	12 months	Access call center, psychological support, weekly diary and self-management advice.	
Walker, P. P. et al. (2018).	I (n = 154), 66% male, mean age 71. C (n = 158), 66% male, mean age 71 (65–76) years.	COPD GOLD II, III, IV, ≥ 60 years, ≥ 1 exacerbation in the past year, ≥ 10 pack-years, ≥ 1 non-pulmonary chronic condition	Evaluate efficacy of home monitoring of lung mechanisms by forced oscillation technique and cardiac parameters on time to hospitalization, HR-QoL and reduce hospital costs.	Within breath respiratory mechanical impedance. CHF patients also measured blood pressure, oxygen saturation, heart rate and body temperature.	1:1 CAU.	No effects were found.	Multi center randomized clinical trial.	9 months	Every 3 months evaluation phone calls. The study nurse received automatic warning if values are worrisome.	The approach is feasible.

* Green: studies with positive effects of the intervention, red: studies without effect of the intervention. ** absence of concomitant severe decompensated diseases; arterial gasometry: pH > 7.35, pO_2_ > 50 mmHg, with O_2_ at a maximum of 3 L/min, and oxygen saturation >90%, pCO_2_ < 55 mmHg; afebrile for more than 48 h; need of administration of bronchodilators a maximum of every 6 h; corticosteroids <40 mg/12 h; chest radiography without new-onset pathology; subjective improvement; and appropriate family environment.
